# Sex differences in the intestinal microbiome: interactions with risk factors for atherosclerosis and cardiovascular disease

**DOI:** 10.1186/s13293-021-00378-z

**Published:** 2021-05-17

**Authors:** Shamon Ahmed, J. David Spence

**Affiliations:** 1 University of British Columbia Faculty of Medicine, Vancouver, British Columbia Canada; 2 Stroke Prevention and Atherosclerosis Research Centre, Robarts Research Institute, Western University, 1400 Western Road, London, Ontario N6G 2V4 Canada

**Keywords:** Atherosclerosis, Cardiovascular risk, Intestinal microbiome, Hypertension, Obesity, Diabetes, Sex differences

## Abstract

**Background:**

There are clearly sex differences in cardiovascular disease. On average, women experience cardiovascular events at an older age, and at any age, women, on average, have less atherosclerotic plaque than men. The role of the human intestinal microbiome in health and disease has garnered significant interest in recent years, and there have been indications of sex differences in the intestinal microbiome. The purpose of this narrative review was to evaluate evidence of sex differences in the interaction between the intestinal microbiome and risk factors for cardiovascular disease. Several studies have demonstrated changes in microbiota composition and metabolic profile as a function of diet, sex hormones, and host metabolism, among other factors. This dysbiosis has consequently been associated with several disease states, including atherosclerosis and cardiovascular disease. In this respect, there is a growing appreciation for the microbiota and its secreted metabolites, including trimethylamine N-oxide (TMAO), derived from intestinal bacterial metabolic pathways involving dietary choline and l-carnitine, as novel risk factors for atherosclerosis and cardiovascular outcomes. Although traditional risk factors for vascular disease have been studied broadly over the years, there exists little research to evaluate interactions of cardiovascular risk factors with a potentially sexually dimorphic intestinal microbiome. This review evaluates the role of sex differences in the composition of the intestinal microbiome, including effects of sex hormones on the microbiome, and the effects of these sex differences on cardiovascular risk factors. Diabetes and obesity exhibit sexual dimorphism, while the data concerning hypertension and dyslipidemia remain inconclusive based on the available literature. In addition, an increased proportion of gram-negative species capable of driving metabolic endotoxemia and a low-grade inflammatory response, as well as decreased numbers of butyrate-producing species, have been observed in relation to traditional vascular risk factors. In this context, circulating SCFAs and TMAO are recognized as key metabolites of the intestinal microbiome that can be readily measured in the blood for the evaluation of metabolic profile.

**Conclusion:**

Novel strategies focused on resolving intestinal dysbiosis as a means to slow progression of atherosclerosis and reduce the risk of cardiovascular disease should be evaluated through a lens of sex differences.

## Introduction

**Fig. 1:**
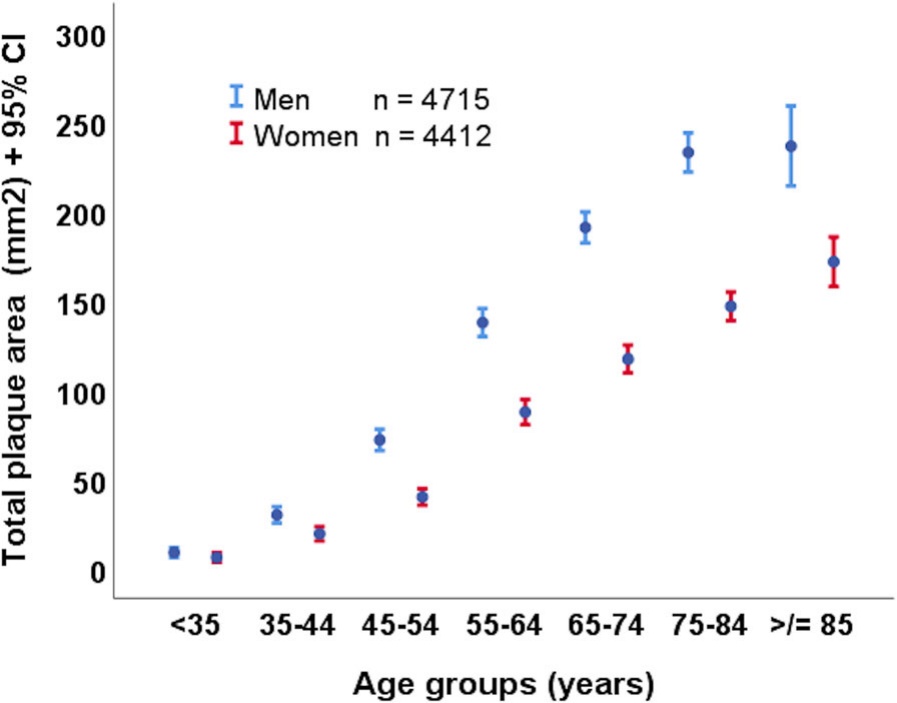
Sex differences in burden of atherosclerosis by age groups. Carotid total plaque area was measured by ultrasound in patients attending vascular prevention clinics at the Stroke Prevention and Atherosclerosis Research Centre (SPARC), Robarts Research Institute, Western University, London, Ontario, Canada. At any age, women on average had less carotid plaque burden than men

There are clear sex difference in atherosclerosis and cardiovascular disease [1]. On average, women experience events such as myocardial infarction at a later age, and on average, have less atherosclerotic plaque than men at any age [2] (Fig. 1). Some recent examples of biological sex differences in atherosclerosis include a report by Ward et al., who in 2018 reported sex differences in the proteomics of atherosclerosis, related to proteins involved in inflammatory responses, response to reactive oxygen species, complement activation, transport and blood coagulation. In 2018, Li et al. [3] reported sex differences in the relationship of fibrinogen to non-calcified and mixed atherosclerotic plaques.

**Fig. 2:**
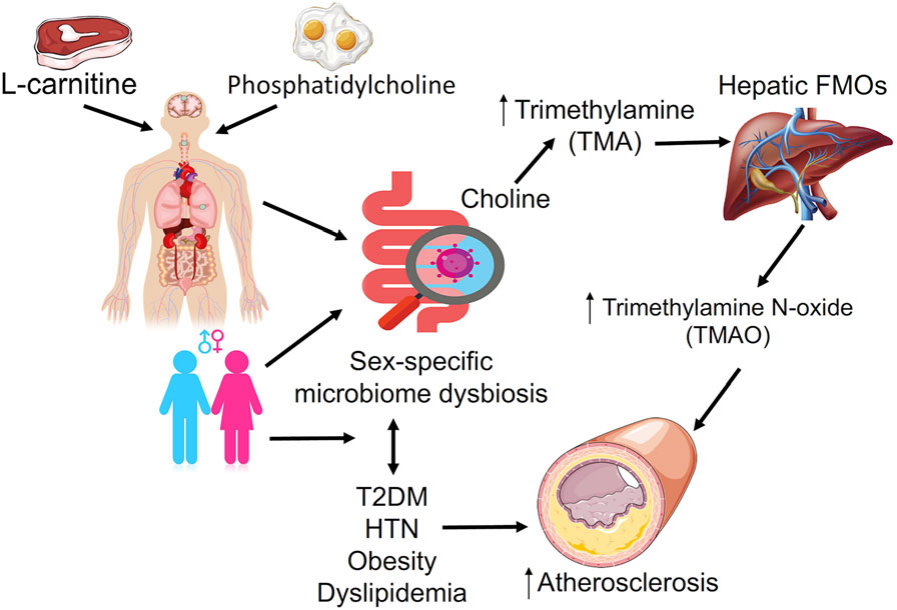
Sex differences in the interaction between the microbiome and risk factors for atherosclerosis and cardiovascular disease. Sex-specific microbiome dysbiosis affects the secretion of metabolites produced by the intestinal microbiome, such as trimethylamine N-oxide (TMAO) derived from dietary intake of phosphatidylcholine and l-carnitine. Such metabolites affect atherosclerosis and cardiovascular risk, through complex interactions with traditional risk factors for atherosclerosis and cardiovascular disease, including diabetes, hypertension, dyslipidemia, and obesity. Flavin mono-oxidase (FMO); hypertension (HTN); type 2 diabetes mellitus (T2DM); trimethylamine N-oxide (TMAO); trimethylamine (TMA)

It has recently become apparent that the intestinal microbiome plays a key role in atherosclerosis and cardiovascular risk. Evidence is emerging that the microbiome affects a number of cardiovascular risk factors, and that there are sex differences in the intestinal microbiome. The purpose of this narrative review is to evaluate the evidence of interactions between sex differences in the intestinal microbiome and cardiovascular risk factors. We focus on sex differences in the composition and metabolic function of the intestinal microbiome, and interactions with traditional risk factors. The following will be addressed: (1) sex differences in the gut microbiome; (2) sex differences with respect to traditional risk factors for atherosclerosis; and (3) the relationship between the human gut microbiome and traditional risk factors for atherosclerosis. Figure 2 illustrates some of the interactions of interest.

The search strategy for the review included a comprehensive search of the English literature using PubMed and Google Scholar. The following search terms were used: “sex AND (intestinal OR gut) AND microbiome”; “sex AND hypertension”; “(intestinal OR gut) AND microbiome AND hypertension”; “(intestinal OR gut) AND microbiome AND hypertension AND sex”; “sex AND diabetes”; “(intestinal OR gut) AND microbiome AND diabetes”; “(intestinal OR gut) AND microbiome AND diabetes AND sex”; “sex AND dyslipidemia”; “(intestinal OR gut) AND microbiome AND dyslipidemia”; “(intestinal OR gut) AND microbiome AND dyslipidemia AND sex”; “sex AND obesity”; “(intestinal OR gut) AND microbiome AND obesity”; “(intestinal OR gut) AND microbiome AND obesity AND sex”; “sex AND cardiovascular AND disease”; “(intestinal OR gut) AND microbiome AND cardiovascular AND disease”; “(intestinal OR gut) AND microbiome AND cardiovascular AND disease AND sex”.

## Background

The study of the human microbiome may have begun some 300 years ago, with the emergence of the microscope, and examination of scrapings from teeth [4]. However, the central role of the intestinal and dental microbiome in health and disease [5] has only been widely appreciated relatively recently [6]. For example, periodontal disease and the dental microbiome have been implicated in atherosclerosis not only through the production of pro-atherogenic metabolites but also directly via systemic inflammation [7]. While there are a number of microbiota (dental, lingual, cutaneous, vaginal, etc.) [8], a key microbiome with regard to metabolic and vascular disease is the intestinal microbiome.

The human gut microbiome consists of trillions of commensal organisms that serve as a barrier and have metabolic functions within the gastrointestinal system [6]. It consists primarily of obligate anaerobes, outnumbering facultative anaerobes and aerobes by up to 100-fold. The two major phyla of bacteria present within the flora are Bacteroidetes and Firmicutes [6, 9]. The primary functions of the intestinal microbiome include digestion, absorption, and production of metabolites from ingested nutrients [10].

Owing to the vastly greater number of microbial organisms and corresponding genes that exist within the gastrointestinal tract as compared to host cells, the human intestinal microbiome has a central role in nutrition, metabolism, and immune function [6, 11, 12]. Among these metabolic functions are the production of vitamins, essential and non-essential amino acids, metabolism of non-digestible carbohydrates such as starches, and biotransformation of bile. The microbiome serves an immuno-protective role by competing with pathogenic organisms for attachment sites in the gut lining, as well as by producing antimicrobial substances. It has also been implicated in signaling to innate immune cells when pathogenic antigens bind to receptors on commensal bacteria, leading to the production of cytokines, peptides, and chemokines that elicit the host immune response [6, 11].

A number of disease states have been explored in relation to the intestinal microbiome, including obesity, inflammatory bowel disease, diabetes, cancer, fatty liver, allergic disease, and CVD. Dysbiosis, which refers to alterations in the normal composition and function of the intestinal microbiome, likely mediates these disease states. Therefore, normalization of the composition and metabolic function of the intestinal microbiome may pose an avenue for therapy [9, 13]. A now widely applied example of this approach is replacement of the intestinal microbiome by fecal microbial transplantation [9], and more recently, with capsules of ecosystems of cultured bacteria [14].

Therefore, the composition of the microbiome and several gut-derived metabolites serve an important role in the development and progression of atherosclerosis [11, 15–19]. This relationship is likely mediated in part by traditional risk factors for atherosclerosis such as obesity, diabetes mellitus, dyslipidemia, and hypertension that have been shown to be associated with dysbiosis [6, 11, 13, 20, 21].

With regard to gut-derived metabolites, several studies have elucidated the importance of the dietary intake of PC (largely from egg yolk) and l-carnitine (largely from red meat) as nutrient precursors of pro-atherogenic molecules such as TMAO. Toxic metabolites of the intestinal microbiome represent novel risk factors for vascular disease. For example, patients with severe atherosclerosis not explained by traditional risk factors have higher plasma levels of toxic metabolites produced by the intestinal microbiome, including TMAO, p-cresyl sulfate, p-cresyl glucuronide, and phenylacetylglutamine, despite no significant differences in dietary intake of nutrient precursors, and no significant differences in renal function [10]. In linear regression, both TMAO and p-cresylsulfate were stronger predictors of carotid plaque burden than several traditional risk factors, including sex, diabetes mellitus, total cholesterol, and diastolic blood pressure.

## Human gut microbiome and atherosclerosis

### Sex differences in intestinal microbiome

As the intestinal microbiome is hypothesized to have a central role in metabolic pathways that drive atherosclerosis, there is growing interest in the presence of sexual dimorphism in the composition of the microbiome. This has important implications for both primary and secondary prevention of vascular disease.

Sex differences in the human intestinal microbiome have been postulated as an explanation for observed epidemiological and phenotypic discrepancies in traditional risk factors for atherosclerosis, including diabetes, hypertension, dyslipidemia, and obesity. For example, in ovariectomized rats with low aerobic capacity, the diversity of the microbiota and specifically the number of Bacteroidetes phylum significantly increased [22]. This reflects the role of sex hormones, including estradiol in modulating the composition of the microbiome. Thus, the increased CVD risk conferred by menopause is likely mediated in part through the intestinal microbiota [23].

Additionally, Haro et al. demonstrated that the proportion of Bacteroides genus was lower in men than women, decreased as BMI increased for men, and remained relatively the same across ranges of BMI for women (*P*<0.001) [24]. Veillonella and Methanobrevibacter genera were more abundant in male fecal samples than in females, while Bilophila was greater in women irrespective of BMI. Furthermore, the microbiota accounted for a statistically significant proportion of the variation in HDL-C, LDL-C, total cholesterol, BMI, and triglycerides.

Markle et al. evaluated sex-hormone driven patterns in autoimmune disease, which display female preponderance [25]. Non-obese diabetic male and female mice experienced elevated testosterone levels when colonized with commensal bacteria relative to germ-free mice. The elevation in testosterone was greater in female mice inoculated with diluted cecal contents from male mice, as compared to un-manipulated female mice. This resulted in a distinct metabolic profile in female gavage recipients that was dissimilar to both un-manipulated male and female mice, suggesting the presence of a sexually dimorphic microbiome that regulated sex hormone production and use. Male to female gavage-recipients were also strongly protected from type 1 diabetes as evidenced by the degree of insulitis, a precursor to overt disease. This effect largely dissipated when the female recipients were treated with the androgen receptor antagonist, flutamide, demonstrating the importance of testosterone signaling in mitigating islet cell inflammation [25].

In a study evaluating the effect of sex hormone perturbations via neonatal androgenization or ovariectomy on the intestinal microbiota of female rats, a lower degree of microbiome diversity was observed in both ovariectomy and androgenized groups. There was however a notable increase in the Firmicutes to Bacteroidetes ratio in the androgenized group [26]. This demonstrated that sex steroid manipulations have a durable impact on the intestinal microbiota, further reflecting the importance of sex differences on its composition. Wang et al. also reported decreased microbiota diversity in male recipients inoculated with fecal bacteria from a donor with a short-term vegetarian and inulin-supplemented diet [27].

This reflects a clear sex-related difference in the composition of the microbiome, and demonstrates that dysbiosis may drive sex differences in disease processes such as atherosclerosis.

### Sex differences in risk factors for atherosclerosis and cardiovascular disease

#### Diabetes mellitus

Diabetes is an established risk factor for atherosclerosis, CAD, and acute MI [28]. A case-control study within the INTERHEART trial by Yusuf et al. demonstrated that diabetes (OR 2.37, PAR 9.9%) accounts for a significant proportion of the risk of MI, irrespective of sex, age, or region of the world [29].

However, there has been much debate regarding sex differences in the risk of atherosclerosis conferred by diabetes [30]. Using the data from the INTERHEART global case-control study, Anand et al. reported that the RR of MI in women who had diabetes was higher than in men (RR 4.26, 95% CI 3.68-4.94 vs. RR 2.67, 95% CI 2.43-2.94) [31]. Women also experienced their first MI at a median age of 65 years, compared to 56 years in men (*P* < 0.0001). This age difference was attributed to higher levels of vascular risk factor levels at younger ages in men.

Several meta-analyses have reported sex differences in the vascular mortality risk conferred by diabetes. The Emerging Risk Factors Collaboration performed a meta-analysis including 698,782 subjects from 102 prospective studies. During 9.8 million years of follow-up, it was found that diabetes was associated with a twofold increased risk of vascular mortality (secondary to occlusive causes), with a greater RR in women than in men [32]. Likewise, the Prospective Studies Collaboration and Asia Pacific Cohort Studies Collaboration reported a meta-analysis that analyzed participant-level data from 980,793 adults. After controlling for major vascular risk factors, including total cholesterol, blood pressure, BMI, and smoking status, diabetes doubled mortality risk among men and tripled risk among women. The RR of occlusive vascular death from diabetes was higher in younger people (aged 35-59) than in older people (70-89), and higher among women across all age groups. However, notably, the absolute excess risk conferred by diabetes was estimated to be similar for men and women despite higher death RRs among women [33]. This demonstrates that lowering of risk factor levels represents an important strategy to reduce occlusive vascular mortality risk in both men and women. However, the excess RR observed among women with diabetes is not accounted for by traditional risk factors and necessitates consideration of other novel risk factors, such as differences in the composition and metabolic function of the intestinal microbiota.

A population-based study in Italy conducted by Ballotari et al. demonstrated that diabetes conferred greater risk of MI in women than in men (IRR 2.58, 95% CI 2.22-3.00 and IRR 1.78, 95% CI 1.60-2.00, *P* < 0.0001) [34]. In a retrospective cohort study by Roche and Wang of 73,783 diabetic individuals in Canada, women with diabetes had a greater risk of all-cause mortality (HR 1.85, 95% CI 1.74-1.96) and CVD hospitalizations (HR 2.57, 95% CI 2.24-2.94) than diabetic men. Among women, those with diabetes demonstrated greater risk (HR 6.54, 95% CI 4.80-8.91 and HR 5.22, 95% CI 4.31-6.33, respectively) than their non-diabetic counterparts, and women in general displayed greater risk than men in any category (HR 3.44, 95% CI 2.47-4.79) and (HR 3.33, 95% CI 2.80-3.95) [35]. Peters et al. performed a systematic review and meta-analysis of 64 cohort studies that included 858,507 individuals with a total of 28,203 coronary events, which revealed that the incident coronary heart disease RR in women with diabetes was 2.82 (95% CI 2.35, 3.38) and 2.16 (95% CI 1.82, 2.56) in men with diabetes [36]. Although it has been asserted that these disparities may be attributed to differences in the use of and compliance with pharmacotherapy between men and women, this is unlikely to explain a 40% greater risk of incident coronary heart disease in women with diabetes.

In the Multi-Ethnic Study of Atherosclerosis (MESA), women with diabetes were less likely to have an LDL-C <130 mg/dl and SBP <130 mmHg than diabetic men [37]. Venegas-Pino et al. reported that male Apo-E-deficient mice developed chronic hyperglycemia, further accelerating atherosclerosis, compared to female mice whose hyperglycemia resolved by 15 weeks of age [38]. This was further explored by castrating male mice, with attenuation of atherosclerotic plaque development. In contrast, atherosclerosis became more advanced in ovariectomized females. This supports the notion that male and female sex hormones are likely central to sex differences in the development of atherosclerosis in the context of diabetes.

#### Hypertension and dyslipidemia

Hypertension and dyslipidemia predispose to atherosclerotic plaque development, particularly at dependent areas at arterial bends, where variations in shear force cause endothelial damage and the accumulation of pro-thrombotic milieu in the local microenvironment [39]. Dyslipidemia provides the substrate for the formation of cholesterol-containing foam cells, while hypertension elicits the necessary endothelial damage for the thrombotic cascade. Despite mechanistic evidence, the relationship between sex, hypertension, and dyslipidemia as it relates to atherosclerosis is not well established.

The INTERHEART global case-control study assessed people in 52 countries; there were 27,098 participants, of whom 6787 were women [31]. Hypertension was more strongly associated with MI among women than in men (OR 2.95, 95% CI 2.66-3.28 vs. OR 2.32, 95% CI 2.16-2.48). Lipids, however, demonstrated similar associations irrespective of sex [31]. In contrast, a systematic review and meta-analysis by Peters et al. [40] compared sex-specific associations between SBP and cardiovascular risk. From 123 prospective cohort studies, including information from 1,197,472 individuals, there was no sex difference in the risk conferred by SBP for stroke or ischemic heart disease. Kren et al. demonstrated increased SBP and levels of serum triglycerides, as well as decreased levels of serum HDL cholesterol in Y consomic rats, suggesting that the Y chromosome may confer increased risk of developing hypertension and dyslipidemia and thus mediate the risk for CVD [41]. Link et al. demonstrated that having 2 X chromosomes versus an X and Y chromosome complement drives sex differences in HDL-C, and not the absence of a Y chromosome [42]. It is conceivable that increased expression of genes escaping X-inactivation in XX mice regulates downstream processes to establish sexual dimorphism in plasma lipid levels. Finally, Wu et al. reported sex differences in cIMT with increased contribution from BMI and LDL to HDL-C ratio in men [43].

Thus, there is no clear consensus on sex-specific associations of atherosclerotic disease with hypertension and dyslipidemia.

#### Obesity

Obesity increases cardiovascular morbidity and mortality, particularly through its association with hypertension and CAD [44, 45]. Within the BMI range of 25–50 kg/m^2^, each 5 kg/m^2^ is associated with ~40% higher stroke mortality [46]. Khan et al. [47] reported a population-based study using pooled individual-level data from adults (baseline age, 20-39, 40-59, and 60-79 years) across 10 large US prospective cohorts, with 3.2 million person-years of follow-up from 1964 to 2015. They studied 190,672 patients, of whom 140,835 (73.9%) were women, free of CVD at baseline. Both being overweight (BMI 25-29) and obesity (BMI > 30) shortened longevity and increased lifetime risk of CVD.

With the growing burden of obesity in North America, understanding sex differences in disease distribution has important implications for prevention and management. Before menopause, women generally have greater vagal than sympathetic tone, and lower levels of total cholesterol and LDL-C than men [48]. Additionally, differences in glucose and lipid metabolism, sex hormones and cytokine production are thought to explain why men are at an increased risk of CVD [48, 49]. This might also explain how disease states such as diabetes, which are characterized by greater levels of inflammation, might predispose to atherosclerosis by abrogating the protective effects of estrogen in maintaining a healthy endothelium, enhancing insulin action, and promoting healthy body fat distribution [49]. Obesity is characterized by an increased risk of diabetes, hypertension and dyslipidemia, and independently associated with CVD [48]. Song et al. reviewed data from 11 prospective cohort trials including 23,629 men and 21,965 women with a median follow-up of 7.9 years, and reported higher CVD mortality among men than women across all anthropometric ranges [48]. This is likely explained by the aforementioned differences in hormone-driven patterns of fat distribution, with men more likely to deposit visceral fat, compared to subcutaneous fat in women [50]. Visceral fat has been associated with greater cardiometabolic risk [51]. These sex-specific differences in CVD mortality were attenuated in obese individuals, particularly those with diabetes, suggesting that obesity confers unfavorable metabolic conditions in both men and women [48]. Furthermore, the age distribution of cardiovascular risk suggests that as women produce less estrogen as they age, they tend to deposit fat in a more “male distribution” intraabdominally, thus explaining the corresponding increase in risk post-menopause [48, 52].

### Human intestinal microbiome and risk factors for atherosclerosis and cardiovascular disease

An important aspect of a sex-based consideration of vascular risk factors for atherosclerosis is whether sex differences in the intestinal microbiome may affect risk factors for atherosclerosis and cardiovascular disease.

#### Diabetes and SCFAs

Several studies have reported that type 2 diabetes mellitus is associated with decreased butyrate-producing species, and an increase in Lactobacillus species [53–57]. Butyrate is a SCFA produced by intestinal microbes from the fermentation of dietary fiber with an important biological role in preventing atherosclerosis [58]. SCFAs such as butyrate, acetate, and propionate have an anti-inflammatory role through the production of Immunoglobulin A and anti-inflammatory cytokines [59], as well as the inhibition of gram-negative translocation across the intestinal luminal barrier [60]. SCFAs also enhance GLP-1 release, which is an incretin hormone involved in decreasing post-prandial blood glucose through inhibition of glucagon release, increased insulin sensitivity, decreased hepatic gluconeogenesis and promotion of satiety [61]. Circulating SCFAs in contrast to fecal SCFAs, have also been shown to be positively associated with fasting GLP-1 concentrations and insulin sensitivity, and negatively associated with whole-body lipolysis, triacylglycerols, and free fatty acid levels [62]. This reflects a direct relationship between circulating SCFAs and metabolic health, and may be an important measurable parameter to evaluate interventions aimed at human metabolism.

Differences in microbiome composition have also been demonstrated in those with diabetes. In a study evaluating fecal bacterial composition by quantitative PCR in 36 men, Firmicutes phylum and Clostridia class were significantly decreased in the diabetic group (*P*=0.03) [53]. Additionally, the ratio of Bacteroidites to Firmicutes and *Bacteroides*-*Prevotella* group to *C. coccoides*-*E. rectale* group correlated positively with plasma glucose in an oral glucose tolerance test, and negatively with BMI, suggesting that gram-negative Bacteroidites and Proteobacteria may contribute to endotoxemia and chronic low-grade inflammation in diabetes [53]. This inflammatory cascade is initiated by lipopolysaccharide in the outer membrane of gram-negative species that translocate across the intestinal luminal barrier. This translocation is promoted by decreased SCFA production [60]. Lipopolysaccharide is a pathogen-associated molecular pattern that serves as an important trigger for the innate immune system [54–56]. The relationship between gram-negative organisms and diabetes is further substantiated by the reduction in insulin sensitivity following vancomycin administration, which resulted in a marked reduction in butyrate-producing organisms [63]. Qin et al. reported that in patients with diabetes, the proportion of opportunistic pathogens was significantly increased, whereas in the non-diabetic group the major phyla were of butyrate-producing microbes [57]. In a bariatric surgery-induced weight loss study, *Faecalibacterium prausnitzii* species were decreased in diabetic subjects and markedly increased in subjects following gastric bypass surgery [64]. *F. prausnitzii* is an anti-inflammatory commensal that inhibits nuclear-factor kappa B activation and the release of pro-inflammatory cytokines such as IL-8 [65]. When live or supernatant *F. prausnitzii* was administered to patients with Crohn’s disease, a reduction in disease severity and resolution of dysbiosis was observed [65]. The microbiome-diabetes interaction may also be mediated through bile acid metabolism. Deoxycholic acid is converted to cholic acid by Clostridium in the large bowel, where cholic acid activates FXR. FXR knockout in mice has been shown to improve glucose tolerance and improve insulin sensitivity [66].

#### Trimethylamine N-oxide (TMAO)

Historically, the proposed link between meat and egg consumption and atherosclerotic disease has been attributed to increased consumption of saturated fat and cholesterol [11]. However, the notion of meta-organismal pathways in which diet-microbe-host interactions contribute to atherosclerotic disease through the production of metabolites and systemic inflammatory response has drawn significant interest recently [11, 67–69]. Central to this pathway are dietary phosphatidyl choline, choline, and l-carnitine [11]. Wang et al. reported that TMAO, produced by hepatic oxidation of trimethylamine (TMA), a metabolite of choline, and betaine, caused atherosclerosis in a murine model, and this was prevented by antibiotics [15]. Koeth et al. reported that mice fed an l-carnitine supplemented diet had high levels of TMAO and twice the aortic root atherosclerotic plaque burden compared to normal chow fed mice, and this could be prevented by antibiotics [19]. This was independent of increases in pro-atherogenic changes in lipids, glucose, lipoproteins, and insulin. When administered antibiotics, plasma trimethylamine and TMAO were significantly reduced and the mice displayed a marked reduction in atherosclerotic plaque burden [19].

Upon ingestion, choline and l-carnitine are metabolized by gut microbes to produce TMA. TMA is absorbed into the portal circulation where two Flavin mono-oxygenase family members (FMO1 and FMO3) within the liver then oxidize TMA to TMAO. TMA is a metabolite of choline, which is derived from foods in the diet such as eggs. FMO3 possesses greater specificity for TMA than FMO1, which is substantiated by increased plasma TMAO in mice with greater expression of FMO3 [10, 24]. Among >4000 patients referred for coronary angiography, plasma TMAO in the top quartile was associated with a 2.5-fold increase in the 3-year risk of stroke, MI, or vascular death [16].

TMAO accounts for a significant proportion of the variation in atherosclerosis [11]. TMAO alters cholesterol and sterol metabolism, upregulating scavenger receptors, which in turn predispose to increased foam cell formation and exacerbate plaque progression [11, 70]. Koeth et al. also demonstrated that TMAO inhibits reverse cholesterol transport, as mice on a TMAO-containing diet had a 35% reduction in cholesterol removal from peripheral macrophages as compared to chow-fed mice (*P*<0.05) [19]. Several bacterial taxa have also been associated with increased plasma TMAO levels, including those belonging to Clostridiaceae and Peptostreptococcaceae families in subjects with omnivorous dietary patterns following an l-carnitine challenge test, suggesting their likely role in the conversion of l-carnitine to TMA [19]. Repeat l-carnitine challenge following administration of broad-spectrum antibiotics virtually suppressed plasma and urine TMAO levels [19]. Thus, microbiota-dependent production of TMA and TMAO through the metabolism of dietary choline, PC, and l-carnitine is associated with increased atherosclerotic risk [11].

Gut-derived metabolites also increase thrombotic potential. The relationship between the gut microbiome and arterial thrombosis was elucidated by Ascher et al., who reported the role of TLR-2 activation by gut microbial ligands in eliciting primary hemostasis at sites of vascular injury via vWF and platelet integrin [71]. Zhu et al. demonstrated a mechanistic link between TMAO and ADP- and thrombin-induced platelet aggregation and adhesion to collagen [72]. This demonstrates that higher plasma TMAO increases vascular thrombosis through both direct and indirect mechanisms, and thereby increases the risk of mortality from stroke or MI in a dose-dependent fashion [72].

Lastly, given that atherosclerosis is a chronic inflammatory state where the innate and adaptive immune system respond to various stimuli, TMAO has been increasingly recognized as an important mediator of systemic inflammation and alterations in immunity [73–75]. Several studies have demonstrated a positive association between plasma TMAO and inflammatory cytokines [76–78]. Chou et al. suggest a correlation between TMAO levels and high sensitivity C-reactive protein (CRP) and IL-1*β* in 81 patients with stable angina [79]. In addition to this, NF-*κ*B pathway has been implicated in atherosclerosis via regulation of pro-inflammatory genes [80, 81]. TMAO has been shown to activate NF-*κ*B to induce the production of pro-inflammatory proteins including cyclooxygenase-2, E-selectin, IL-6, and intracellular adhesion molecule-1 [82]. This relationship is further substantiated by the increased expression of NF-*κ*B-mediated inflammatory genes in aortic endothelium in mice fed a choline diet with elevated TMAO levels [82]. Thus, the association between TMAO and atherosclerosis and cardiovascular risk is now well established [11].

#### Therapeutic aim

The importance of gut microbes in nutrient metabolism and atherosclerosis has generated interest in novel approaches that aim to reduce TMA conversion to TMAO, the use of high-fiber diets to decrease TMA precursors, and the maintenance of an optimal gut microbial composition [83]. A proposed mechanism for how high-fiber diets decrease TMA precursors is via activation of epithelial adenosine monophosphate-activated protein kinase, which inhibits TMA lyase activity and increases expression of SCFAs including acetate and butyrate [84]. Fecal microbial transplantation for this purpose is currently under study at our research center; it is hoped that identifying bacteria associated with reduce plasma levels of TMAO and p-cresylsulfate will permit identification of bacteria to be included in an “ecosystem therapeutic” of cultured bacteria, as has previously been used to treat infection with *Helicobacter pylori* [14]. Non-lethal inhibitors of trimethylamine lyase, which catalyzes the conversion of choline to TMA, are also being explored as therapies for atherosclerosis [85, 86].

#### Hypertension

Hypertension and the intestinal microbiome drew interest following the report by Honour in 1982, which demonstrated that rats administered antibiotics along with corticosteroids experienced a smaller increase in blood pressure than those administered corticosteroids alone [87]. This gave credence to the argument that the microbiome was involved in steroidal hypertension. Yang et al. reported decreased microbial diversity and an increase in Firmicutes to Bacteroidetes ratio in SPH rats compared to controls [88]. Additionally, SPH rats had increased lactate-producing microbes such as Streptococcus and Turicibacter, and decreased butyrate-producing microbes. In contrast, control rats had increased proportions of butyrate-producing organisms such as *Coprococcus* and *Pseudobutyrivibrio*. Following treatment with minocycline for 4 weeks, MAP was significantly reduced in Angiotensin II-infused rats (24-h MAP: 124 ± 2 mmHg vs 168 ± 2 mmHg). Likewise, transfer of microbiota into germ-free mice resulted in greater endothelial dysfunction [89]. When these germ-free mice were infused with Angiotensin-II, a marked reduction in reactive oxygen species production, monocyte chemoattractant protein-1, inducible nitric oxide synthase, and NADPH oxidase subunit Nox2, was observed relative to control mice. This reflects a microbiota-dependent response to Angiotensin-II, and implicates commensal microbes in vascular dysfunction and hypertension [89]. Engevik et al. reported alterations in intestinal microbiota, with increased populations of Bacteroidetes compared to Firmicutes in regions of the small colon deficient in NHE3 [90]. Additionally, Li et al. reported that in genetically deficient NHE3 mice, MAP and SBP increases were attenuated upon infusion of angiotensin II as compared to control [91]. As NHE3 plays an important role in salt and water absorption both in the intestinal and the kidneys, and since excessive salt intake is associated with hypertension, it is reasonable to infer that the intestinal microbiome may mediate hypertension through the action of ion channels. Li et al. reported decreased microbial diversity and richness, and increased populations of Prevotella and Klebsiella genera in pre-hypertensive and hypertensive human subjects [92]. In contrast, healthy controls had increased populations of *Faecalibacterium*, *Oscillibacter*, *Roseburia*, *Bifidobacterium*, *Coprococcus*, and *Butyrivibrio. Roseburia and Faecalibacterium* are two butyrate producing organisms that have been negatively associated with inflammatory bowel disease, suggesting their role in health and disease [65, 93]. When germ-free mice were inoculated with fecal samples from hypertensive human subjects, the mice exhibited greater systolic, diastolic, and mean blood pressures as compared to controls (*P*<0.05). This is in line with previous work demonstrating blood pressure attenuation through the use of antibiotics and probiotics [88, 89, 94–96]. Another mechanism by which intestinal bacteria may be implicated in hypertension is through the production of microbial SCFAs that act on G-coupled protein receptors to activate sympathetic activity and induce renin secretion [97].

#### Obesity

With regard to obesity, a prospective trial conducted by Collado et al. demonstrated distinct human intestinal microbiota composition among women as a function of weight and BMI during pregnancy [98]. Higher weight correlated with higher concentrations of Bacteroides, Clostridium, and Staphylococcus. Similarly, due to the apparent sexual dimorphism between obesity and chronic disease, Nickelson et al. compared weight-matched obese male and female mice to determine if the sex-dependent health benefits remain when body weight is similar [99]. In comparing weight-matched obese male and female mice receiving a high-fat diet, it was found that female mice exhibited greater adiposity. Despite this, female mice were more glucose tolerant, likely due to increased adiponectin and decreased oxidative stress in the presence of estrogen. Turnbaugh et al. reported that obese mice have significant differences in the populations of two bacterial species: Firmicutes and Bacteroidetes [100]. Compared to lean mice, the obese microbiome was able to extract more energy from the diet, and upon colonization of germ-free mice with the obese microbiome, the mice had an increase in total body fat. Vrieze et al. similarly reported that colonization of recipients with microbiota from lean donors increased insulin sensitivity (median rate of glucose disappearance changed from 26.2 to 45.3 mumol/kg/min; *P* < .05) [56]. Mongraw-Chaffin et al. reported that sex hormones are significantly associated with adiposity, and the associations of androgens differ qualitatively by sex. This heterogeneity may help explain the complexity of the contribution of sex hormones to sex differences in CVD [101, 102]. In a mouse model of gastric bypass, increased populations of Gammaproteobacteria (*Escherichia*) and Verrucomicrobia (*Akkermansia*) were observed, independent of changes in weight and calorie intake [103]. Inoculation of germ-free mice with microbiota from gastric bypass-treated mice resulted in decreased adiposity and weight loss [103]. These compositional changes are similar to those exhibited by human subjects following gastric bypass. For example, Furet et al. reported lower Bacteroides/Prevotella group in obese subjects, and a corresponding increase following bariatric surgery. Likewise, at 3 months post-bypass, *Escherichia coli* exhibited a significant increase as compared to control [64]. In contrast, levels of Bifidobacterium and Lactobacillus/Leuconostoc/Pediococcus groups decreased at 3 and 6 months following surgery. These data collectively demonstrate that obesity is associated with characteristic changes in the microbiome.

## Conclusion

Enhanced insight into meta-organismal pathways that drive atherosclerosis has drawn interest toward patient characteristics such as sex, as it has important implications for the management of vascular diseases. In this context, diabetes and obesity appear to demonstrate sexual dimorphism, while the data concerning hypertension and dyslipidemia are less conclusive. A greater proportion of gram-negative species with a decreased capacity for butyrate-production have also been observed in relation to traditional vascular risk factors [58]. In addition to this, circulating SCFAs and TMAO are important novel risk factors for atherosclerosis that can be measured in the blood, and present an important opportunity for the assessment and management of dysbiosis, particularly through the use of high fiber diets which have been shown to increase circulating SCFAs and decrease TMAO [62, 84]. Male and female sex hormones also play an important role in the composition and metabolic function of the microbiome, and thus have differential effects on disease incidence and phenotype. Finally, sex differences clearly exist in established risk factors for atherosclerosis, and further investigation is necessary to assess whether differential responses in the context of similar microbiome composition may implicate other host factors in the risk for atherosclerosis. Nevertheless, novel treatment strategies for atherosclerosis focused on dysbiosis require sex-specific consideration, as sex differences have important effects on several established vascular risk factors.

### Perspectives and significance

This study highlights the role of a sexually dimorphic microbiome in mediating the risk for atherosclerotic disease, both through traditional risk factors, novel metabolites such as SCFAs and TMAO, and via chronic systemic inflammation. Pre-biotic and probiotic interventions such as dietary fiber, TMA lyase inhibitors, and fecal transplantation aimed at treating dysbiosis in the context of atherosclerosis should therefore be viewed through a sex-specific lens. Further study is required to elucidate the role of other host factors in mediating sex-specific differences in disease incidence and phenotype, when such differences cannot be explained by microbiome composition and function.

## Data Availability

An EndNote reference database is available on request.

## Abbreviations

ADP: Adenosine diphosphate
BMI: Body Mass Index
CAD: Coronary artery disease
CI: Confidence interval
cIMT: Carotid intimal medial thickness
CRP: C-reactive protein
CVD: Cardiovascular disease
FMO1: Flavin monooxygenase-1
FMO3: Flavin monooxygenase-3
FXR: Farnesoid X receptor
GLP-1: Glucagon-like peptide-1
HDL-C: High-density lipoprotein-Cholesterol
HR: Hazard ratio
IgA: Immunoglobulin A
IL-8: Interleukin-8
IRR: Incidence rate ratios
LDL-C: Low-density lipoprotein-Cholesterol
MAP: Mean arterial blood pressure
MESA: Multi-ethnic study of atherosclerosis
MI: Myocardial infarction
NADPH: Nicotinamide adenine dinucleotide phosphate
NHE3: Na^+^/H^+^-exchanger isoform 3
Nox2: NADPH oxidase 2
OR: Odds ratio
PAR: Population attributable risk
PC: Phosphatidylcholine
PCR: Polymerase chain reaction
RR: Relative risk
SBP: Systolic blood pressure
SCFA: Short chain fatty acid
SPH: Spontaneously hypertensive
TLR-2: Toll-like receptor 2
TMA: Trimethylamine
TMAO: Trimethylamine oxidase
vWF: Von Willebrand factor

## References

[1] Spence JD, Pilote L (2015). Importance of sex and gender in atherosclerosis and cardiovascular disease. Atherosclerosis..

[2] Iemolo F, Martiniuk A, Steinman DA, Spence JD (2004). Sex differences in carotid plaque and stenosis. Stroke..

[3] Li T, Wang F, Peng R, Pei S, Hou Z, Lu B, Cong X, Chen X (2018). Sex-related differences in the association between plasma fibrinogen and non-calcified or mixed coronary atherosclerotic plaques. Biol Sex Differ.

[4] Relman DA (2015). The human microbiome and the future practice of medicine. JAMA..

[5] Clemente JC, Ursell LK, Parfrey LW, Knight R (2012). The impact of the gut microbiota on human health: an integrative view. Cell..

[6] Bull MJ, Plummer NT (2014). Part 1: The human gut microbiome in health and disease. Integr Med (Encinitas, Calif).

[7] Fåk F, Tremaroli V, Bergström G, Bäckhed F (2015). Oral microbiota in patients with atherosclerosis. Atherosclerosis..

[8] Young LH, Viscoli CM, Curtis JP, Inzucchi SE, Schwartz GG, Lovejoy AM, Furie KL, Gorman MJ, Conwit R, Abbott JD, Jacoby DL, Kolansky DM, Pfau SE, Ling FS, Kernan WN, IRIS Investigators (2017). Cardiac outcomes after ischemic stroke or transient ischemic attack: effects of pioglitazone in patients with insulin resistance without diabetes mellitus. Circulation..

[9] Gupta S, Allen-Vercoe E, Petrof EO (2016). Fecal microbiota transplantation: in perspective. Ther Adv Gastroenterol.

[10] Bogiatzi C, Gloor G, Allen-Vercoe E, Reid G, Wong RG, Urquhart BL, Dinculescu V, Ruetz KN, Velenosi TJ, Pignanelli M, Spence JD (2018). Metabolic products of the intestinal microbiome and extremes of atherosclerosis. Atherosclerosis..

[11] Brown JM, Hazen SL (2014). Metaorganismal nutrient metabolism as a basis of cardiovascular disease. Curr Opin Lipidol.

[12] Qin J, Li R, Raes J, Arumugam M, Burgdorf KS, Manichanh C (2010). A human gut microbial gene catalogue established by metagenomic sequencing. Nature..

[13] Vinje S, Stroes E, Nieuwdorp M, Hazen SL (2014). The gut microbiome as novel cardio-metabolic target: the time has come!. Eur Heart J.

[14] Petrof EO, Gloor GB, Vanner SJ, Weese SC, Carter D, Daigneaul MC, Brown EM (2013). Stool substitute transplant therapy for the eradication of *Clostridium difficile* infection: ‘RePOOPulating’ the gut. Microbiome..

[15] Wang Z, Klipfell E, Bennett BJ, Koeth R, Levison BS, Dugar B (2011). Gut flora metabolism of phosphatidylcholine promotes cardiovascular disease. Nature..

[16] Tang WHW, Wang Z, Levison BS, Koeth RA, Britt EB, Fu X, Wu Y, Hazen SL (2013). Intestinal microbial metabolism of phosphatidylcholine and cardiovascular risk. N Engl J Med.

[17] Ussher JR, Lopaschuk GD, Arduini A (2013). Gut microbiota metabolism of L-carnitine and cardiovascular risk. Atherosclerosis..

[18] Koeth RA, Levison BS, Culley MK, Buffa JA, Wang Z, Gregory JC, Org E, Wu Y, Li L, Smith JD, Tang WHW, DiDonato JA, Lusis AJ, Hazen SL (2014). gamma-Butyrobetaine is a proatherogenic intermediate in gut microbial metabolism of L-carnitine to TMAO. Cell Metab.

[19] Koeth RA, Wang Z, Levison BS, Buffa JA, Org E, Sheehy BT, Britt EB, Fu X, Wu Y, Li L, Smith JD, DiDonato JA, Chen J, Li H, Wu GD, Lewis JD, Warrier M, Brown JM, Krauss RM, Tang WHW, Bushman FD, Lusis AJ, Hazen SL (2013). Intestinal microbiota metabolism of L-carnitine, a nutrient in red meat, promotes atherosclerosis. Nat Med.

[20] Singh V, Yeoh BS, Vijay-Kumar M (2016). Gut microbiome as a novel cardiovascular therapeutic target. Curr Opin Pharmacol.

[21] Org E, Mehrabian M, Lusis AJ (2015). Unraveling the environmental and genetic interactions in atherosclerosis: central role of the gut microbiota. Atherosclerosis..

[22] Cox-York KA, Sheflin AM, Foster MT, Gentile CL, Kahl A, Koch LG (2015). Ovariectomy results in differential shifts in gut microbiota in low versus high aerobic capacity rats. Phys Rep.

[23] Tims S, Derom C, Jonkers DM, Vlietinck R, Saris WH, Kleerebezem M, de Vos WM, Zoetendal EG (2013). Microbiota conservation and BMI signatures in adult monozygotic twins. ISME J.

[24] Haro C, Rangel-Zuniga OA, Alcala-Diaz JF, Gomez-Delgado F, Perez-Martinez P, Delgado-Lista J (2016). Intestinal microbiota is influenced by gender and body mass index. PLoS One.

[25] Markle JG, Frank DN, Mortin-Toth S, Robertson CE, Feazel LM, Rolle-Kampczyk U (2013). Sex differences in the gut microbiome drive hormone-dependent regulation of autoimmunity. Science (New York, NY).

[26] Moreno-Indias I, Sanchez-Alcoholado L, Sanchez-Garrido MA, Martin-Nunez GM, Perez-Jimenez F, Tena-Sempere M (2016). Neonatal androgen exposure causes persistent gut microbiota dysbiosis related to metabolic disease in adult female rats. Endocrinology..

[27] Wang JJ, Wang J, Pang XY, Zhao LP, Tian L, Wang XP (2016). Sex differences in colonization of gut microbiota from a man with short-term vegetarian and inulin-supplemented diet in germ-free mice. Sci Rep.

[28] Madonna R, Balistreri CR, De Rosa S, Muscoli S, Selvaggio S, Selvaggio G (2019). Impact of sex differences and diabetes on coronary atherosclerosis and ischemic heart disease. J Clin Med.

[29] Yusuf S, Hawken S, Ounpuu S, Dans T, Avezum A, Lanas F (2004). Effect of potentially modifiable risk factors associated with myocardial infarction in 52 countries (the INTERHEART study): case-control study. Lancet (London, England).

[30] Kautzky-Willer A, Harreiter J, Pacini G (2016). Sex and gender differences in risk, pathophysiology and complications of type 2 diabetes mellitus. Endocr Rev.

[31] Anand SS, Islam S, Rosengren A, Franzosi MG, Steyn K, Yusufali AH, Keltai M, Diaz R, Rangarajan S, Yusuf S, INTERHEART Investigators (2008). Risk factors for myocardial infarction in women and men: insights from the INTERHEART study. Eur Heart J.

[32] Sarwar N, Gao P, Seshasai SR, Gobin R, Kaptoge S, Di Angelantonio E (2010). Diabetes mellitus, fasting blood glucose concentration, and risk of vascular disease: a collaborative meta-analysis of 102 prospective studies. Lancet (London, England).

[33] Prospective Studies Collaboration and Asia Pacific Cohort Studies Collaboration. Sex-specific relevance of diabetes to occlusive vascular and other mortality: a collaborative meta-analysis of individual data from 980 793 adults from 68 prospective studies. Lancet Diabetes Endocrinol. 2018;6(7):538–46. 10.1016/S2213-8587(18)30079-2 PMC6008496 29752194

[34] Ballotari P, Venturelli F, Greci M, Giorgi Rossi P, Manicardi V (2017). Sex Differences in the effect of type 2 diabetes on major cardiovascular diseases: results from a population-based study in Italy. Int J Endocrinol.

[35] Roche MM, Wang PP (2013). Sex differences in all-cause and cardiovascular mortality, hospitalization for individuals with and without diabetes, and patients with diabetes diagnosed early and late. Diabetes Care.

[36] Peters SA, Huxley RR, Woodward M (2014). Diabetes as risk factor for incident coronary heart disease in women compared with men: a systematic review and meta-analysis of 64 cohorts including 858,507 individuals and 28,203 coronary events. Diabetologia..

[37] Winston GJ, Barr RG, Carrasquillo O, Bertoni AG, Shea S (2009). Sex and racial/ethnic differences in cardiovascular disease risk factor treatment and control among individuals with diabetes in the Multi-Ethnic Study of Atherosclerosis (MESA). Diabetes Care.

[38] Venegas-Pino DE, Wang PW, Stoute HK, Singh-Pickersgill NA, Hong BY, Khan MI, Shi Y, Werstuck GH (2016). Sex-specific differences in an ApoE(−/−):Ins2(+/Akita) mouse model of accelerated atherosclerosis. Am J Pathol.

[39] Singh RB, Mengi SA, Xu YJ, Arneja AS, Dhalla NS (2002). Pathogenesis of atherosclerosis: a multifactorial process. Exp Clin Cardiol.

[40] Peters SA, Huxley RR, Woodward M (2013). Comparison of the sex-specific associations between systolic blood pressure and the risk of cardiovascular disease: a systematic review and meta-analysis of 124 cohort studies, including 1.2 million individuals. Stroke..

[41] Kren V, Qi N, Krenova D, Zidek V, Sladka M, Jachymova M (2001). Y-chromosome transfer induces changes in blood pressure and blood lipids in SHR. Hypertension (Dallas, Tex: 1979).

[42] Link JC, Chen X, Prien C, Borja MS, Hammerson B, Oda MN, Arnold AP, Reue K (2015). Increased high-density lipoprotein cholesterol levels in mice with XX versus XY sex chromosomes. Arterioscler Thromb Vasc Biol.

[43] Wu TW, Hung CL, Liu CC, Wu YJ, Wang LY, Yeh HI (2017). Associations of cardiovascular risk factors with carotid intima-media thickness in middle-age adults and elders. J Atheroscler Thromb.

[44] Rocha VZ, Libby P (2009). Obesity, inflammation, and atherosclerosis. Nat Rev Cardiol.

[45] Wang Z, Nakayama T (2010). Inflammation, a link between obesity and cardiovascular disease. Mediat Inflamm.

[46] Prospective Studies C, Whitlock G, Lewington S, Sherliker P, Clarke R, Emberson J (2009). Body-mass index and cause-specific mortality in 900 000 adults: collaborative analyses of 57 prospective studies. Lancet..

[47] Khan SS, Ning H, Wilkins JT, Allen N, Carnethon M, Berry JD, Sweis RN, Lloyd-Jones DM (2018). Association of body mass index with lifetime risk of cardiovascular disease and compression of morbidity. JAMA Cardiol.

[48] Song X, Tabak AG, Zethelius B, Yudkin JS, Soderberg S, Laatikainen T (2014). Obesity attenuates gender differences in cardiovascular mortality. Cardiovasc Diabetol.

[49] Stubbins RE, Holcomb VB, Hong J, Nunez NP (2012). Estrogen modulates abdominal adiposity and protects female mice from obesity and impaired glucose tolerance. Eur J Nutr.

[50] Varlamov O, Bethea CL, Roberts CT (2014). Sex-specific differences in lipid and glucose metabolism. Front Endocrinol.

[51] Sironi AM, Petz R, De Marchi D, Buzzigoli E, Ciociaro D, Positano V (2012). Impact of increased visceral and cardiac fat on cardiometabolic risk and disease. Diabet Med.

[52] Tamargo J, Rosano G, Walther T, Duarte J, Niessner A, Kaski JC, Ceconi C, Drexel H, Kjeldsen K, Savarese G, Torp-Pedersen C, Atar D, Lewis BS, Agewall S (2017). Gender differences in the effects of cardiovascular drugs. Eur Heart J Cardiovasc Pharmacother.

[53] Larsen N, Vogensen FK, van den Berg FW, Nielsen DS, Andreasen AS, Pedersen BK (2010). Gut microbiota in human adults with type 2 diabetes differs from non-diabetic adults. PLoS One.

[54] Cani PD, Amar J, Iglesias MA, Poggi M, Knauf C, Bastelica D, Neyrinck AM, Fava F, Tuohy KM, Chabo C, Waget A, Delmee E, Cousin B, Sulpice T, Chamontin B, Ferrieres J, Tanti JF, Gibson GR, Casteilla L, Delzenne NM, Alessi MC, Burcelin R (2007). Metabolic endotoxemia initiates obesity and insulin resistance. Diabetes..

[55] Cani PD, Bibiloni R, Knauf C, Waget A, Neyrinck AM, Delzenne NM, Burcelin R (2008). Changes in gut microbiota control metabolic endotoxemia-induced inflammation in high-fat diet-induced obesity and diabetes in mice. Diabetes..

[56] Vrieze A, Van Nood E, Holleman F, Salojarvi J, Kootte RS, Bartelsman JF (2012). Transfer of intestinal microbiota from lean donors increases insulin sensitivity in individuals with metabolic syndrome. Gastroenterology.

[57] Qin J, Li Y, Cai Z, Li S, Zhu J, Zhang F, Liang S, Zhang W, Guan Y, Shen D, Peng Y, Zhang D, Jie Z, Wu W, Qin Y, Xue W, Li J, Han L, Lu D, Wu P, Dai Y, Sun X, Li Z, Tang A, Zhong S, Li X, Chen W, Xu R, Wang M, Feng Q, Gong M, Yu J, Zhang Y, Zhang M, Hansen T, Sanchez G, Raes J, Falony G, Okuda S, Almeida M, LeChatelier E, Renault P, Pons N, Batto JM, Zhang Z, Chen H, Yang R, Zheng W, Li S, Yang H, Wang J, Ehrlich SD, Nielsen R, Pedersen O, Kristiansen K, Wang J (2012). A metagenome-wide association study of gut microbiota in type 2 diabetes. Nature..

[58] Aw W, Fukuda S (2018). Understanding the role of the gut ecosystem in diabetes mellitus. J Diabetes Invest.

[59] Tan J, McKenzie C, Potamitis M, Thorburn AN, Mackay CR, Macia L (2014). The role of short-chain fatty acids in health and disease. Adv Immunol.

[60] Amar J, Chabo C, Waget A, Klopp P, Vachoux C, Bermudez-Humaran LG (2011). Intestinal mucosal adherence and translocation of commensal bacteria at the early onset of type 2 diabetes: molecular mechanisms and probiotic treatment. EMBO Mol Med.

[61] Thomas C, Gioiello A, Noriega L, Strehle A, Oury J, Rizzo G, Macchiarulo A, Yamamoto H, Mataki C, Pruzanski M, Pellicciari R, Auwerx J, Schoonjans K (2009). TGR5-mediated bile acid sensing controls glucose homeostasis. Cell Metab.

[62] Müller M, Hernández MAG, Goossens GH, Reijnders D, Holst JJ, Jocken JWE, van Eijk H, Canfora EE, Blaak EE (2019). Circulating but not faecal short-chain fatty acids are related to insulin sensitivity, lipolysis and GLP-1 concentrations in humans. Sci Rep.

[63] Vrieze A, Out C, Fuentes S, Jonker L, Reuling I, Kootte RS, van Nood E, Holleman F, Knaapen M, Romijn JA, Soeters MR, Blaak EE, Dallinga-Thie GM, Reijnders D, Ackermans MT, Serlie MJ, Knop FK, Holst JJ, van der Ley C, Kema IP, Zoetendal EG, de Vos WM, Hoekstra JBL, Stroes ES, Groen AK, Nieuwdorp M (2014). Impact of oral vancomycin on gut microbiota, bile acid metabolism, and insulin sensitivity. J Hepatol.

[64] Furet JP, Kong LC, Tap J, Poitou C, Basdevant A, Bouillot JL, Mariat D, Corthier G, Dore J, Henegar C, Rizkalla S, Clement K (2010). Differential adaptation of human gut microbiota to bariatric surgery-induced weight loss: links with metabolic and low-grade inflammation markers. Diabetes..

[65] Sokol H, Pigneur B, Watterlot L, Lakhdari O, Bermudez-Humaran LG, Gratadoux JJ, Blugeon S, Bridonneau C, Furet JP, Corthier G, Grangette C, Vasquez N, Pochart P, Trugnan G, Thomas G, Blottiere HM, Dore J, Marteau P, Seksik P, Langella P (2008). Faecalibacterium prausnitzii is an anti-inflammatory commensal bacterium identified by gut microbiota analysis of Crohn disease patients. Proc Natl Acad Sci U S A.

[66] Ryan KK, Tremaroli V, Clemmensen C, Kovatcheva-Datchary P, Myronovych A, Karns R, Wilson-Pérez HE, Sandoval DA, Kohli R, Bäckhed F, Seeley RJ (2014). FXR is a molecular target for the effects of vertical sleeve gastrectomy. Nature..

[67] Brown JM, Hazen SL (2018). Microbial modulation of cardiovascular disease. Nat Rev Microbiol.

[68] Drosos I, Tavridou A, Kolios G (2015). New aspects on the metabolic role of intestinal microbiota in the development of atherosclerosis. Metab Clin Exp.

[69] Spector R (2016). New insight into the dietary cause of atherosclerosis: implications for pharmacology. J Pharmacol Exp Ther.

[70] Canyelles M, Tondo M, Cedo L, Farras M, Escola-Gil JC, Blanco-Vaca F (2018). Trimethylamine N-oxide: a link among diet, gut microbiota, gene regulation of liver and intestine cholesterol homeostasis and HDL function. Int J Mol Sci.

[71] Ascher S, Reinhardt C (2018). The gut microbiota: an emerging risk factor for cardiovascular and cerebrovascular disease. Eur J Immunol.

[72] Zhu W, Gregory JC, Org E, Buffa JA, Gupta N, Wang Z, Li L, Fu X, Wu Y, Mehrabian M, Sartor RB, McIntyre TM, Silverstein RL, Tang WHW, DiDonato JA, Brown JM, Lusis AJ, Hazen SL (2016). Gut microbial metabolite TMAO enhances platelet hyperreactivity and thrombosis risk. Cell..

[73] Ross R (1999). Atherosclerosis--an inflammatory disease. New Engl J Med.

[74] Libby P, Ridker PM, Maseri A (2002). Inflammation and atherosclerosis. Circulation..

[75] Tuttolomondo A, Di Raimondo D, Pecoraro R, Arnao V, Pinto A, Licata G (2012). Atherosclerosis as an inflammatory disease. Curr Pharm Des.

[76] Boutagy NE, Neilson AP, Osterberg KL, Smithson AT, Englund TR, Davy BM (2015). Short-term high-fat diet increases postprandial trimethylamine-N-oxide in humans. Nutr Res (New York, NY).

[77] Chen K, Zheng X, Feng M, Li D, Zhang H (2017). Gut microbiota-dependent metabolite trimethylamine N-oxide contributes to cardiac dysfunction in Western diet-induced obese mice. Front Physiol.

[78] Rohrmann S, Linseisen J, Allenspach M, von Eckardstein A, Müller D (2016). Plasma concentrations of trimethylamine-N-oxide are directly associated with dairy food consumption and low-grade inflammation in a German adult population. J Nutr.

[79] Chou RH, Chen CY, Chen IC, Huang HL, Lu YW, Kuo CS, Chang CC, Huang PH, Chen JW, Lin SJ (2019). Trimethylamine N-oxide, circulating endothelial progenitor cells, and endothelial function in patients with stable angina. Sci Rep.

[80] Tak PP, Firestein GS (2001). NF-kappaB: a key role in inflammatory diseases. J Clin Invest.

[81] Baker RG, Hayden MS, Ghosh S (2011). NF-κB, inflammation, and metabolic disease. Cell Metab.

[82] Seldin MM, Meng Y, Qi H, Zhu W, Wang Z, Hazen SL (2016). Trimethylamine N-oxide promotes vascular inflammation through signaling of mitogen-activated protein kinase and nuclear factor-κB. J Am Heart Assoc.

[83] Anbazhagan AN, Priyamvada S, Priyadarshini M (2017). Gut microbiota in vascular disease: therapeutic target?. Curr Vasc Pharmacol.

[84] Li Q, Wu T, Liu R, Zhang M, Wang R. Soluble dietary fiber reduces trimethylamine metabolism via gut microbiota and co-regulates host AMPK pathways. Mol Nutr Food Res. 2017;61(12). 10.1002/mnfr.201700473. 10.1002/mnfr.201700473 28884952

[85] Wang Z, Roberts AB, Buffa JA, Levison BS, Zhu W, Org E, Gu X, Huang Y, Zamanian-Daryoush M, Culley MK, DiDonato AJ, Fu X, Hazen JE, Krajcik D, DiDonato JA, Lusis AJ, Hazen SL (2015). Non-lethal inhibition of gut microbial trimethylamine production for the treatment of atherosclerosis. Cell..

[86] Roberts AB, Gu X, Buffa JA, Hurd AG, Wang Z, Zhu W (2018). Development of a gut microbe–targeted nonlethal therapeutic to inhibit thrombosis potential. Nat Med.

[87] Honour J (1982). The possible involvement of intestinal bacteria in steroidal hypertension. Endocrinology..

[88] Yang T, Santisteban MM, Rodriguez V, Li E, Ahmari N, Carvajal JM (2015). Gut dysbiosis is linked to hypertension. Hypertension (Dallas, Tex: 1979).

[89] Karbach SH, Schonfelder T, Brandao I, Wilms E, Hormann N, Jackel S (2016). Gut microbiota promote angiotensin II-induced arterial hypertension and vascular dysfunction. J Am Heart Assoc.

[90] Engevik MA, Aihara E, Montrose MH, Shull GE, Hassett DJ, Worrell RT (2013). Loss of NHE3 alters gut microbiota composition and influences Bacteroides thetaiotaomicron growth. Am J Physiol Gastrointest Liver Physiol.

[91] Li XC, Shull GE, Miguel-Qin E, Chen F, Zhuo JL (2015). Role of the Na+/H+ exchanger 3 in angiotensin II-induced hypertension in NHE3-deficient mice with transgenic rescue of NHE3 in small intestines. Phys Rep.

[92] Li J, Zhao F, Wang Y, Chen J, Tao J, Tian G, Wu S, Liu W, Cui Q, Geng B, Zhang W, Weldon R, Auguste K, Yang L, Liu X, Chen L, Yang X, Zhu B, Cai J (2017). Gut microbiota dysbiosis contributes to the development of hypertension. Microbiome..

[93] Machiels K, Joossens M, Sabino J, De Preter V, Arijs I, Eeckhaut V (2014). A decrease of the butyrate-producing species Roseburia hominis and Faecalibacterium prausnitzii defines dysbiosis in patients with ulcerative colitis. Gut..

[94] Khalesi S, Sun J, Buys N, Jayasinghe R (2014). Effect of probiotics on blood pressure: a systematic review and meta-analysis of randomized, controlled trials. Hypertension (Dallas, Tex: 1979).

[95] Gomez-Guzman M, Toral M, Romero M, Jimenez R, Galindo P, Sanchez M (2015). Antihypertensive effects of probiotics Lactobacillus strains in spontaneously hypertensive rats. Mol Nutr Food Res.

[96] Qi Y, Aranda JM, Rodriguez V, Raizada MK, Pepine CJ (2015). Impact of antibiotics on arterial blood pressure in a patient with resistant hypertension - a case report. Int J Cardiol.

[97] Pluznick JL (2017). Microbial short-chain fatty acids and blood pressure regulation. Curr Hypertens Rep.

[98] Collado MC, Isolauri E, Laitinen K, Salminen S (2008). Distinct composition of gut microbiota during pregnancy in overweight and normal-weight women. Am J Clin Nutr.

[99] Nickelson KJ, Stromsdorfer KL, Pickering RT, Liu TW, Ortinau LC, Keating AF (2012). A comparison of inflammatory and oxidative stress markers in adipose tissue from weight-matched obese male and female mice. Exp Diabetes Res.

[100] Turnbaugh PJ, Ley RE, Mahowald MA, Magrini V, Mardis ER, Gordon JI (2006). An obesity-associated gut microbiome with increased capacity for energy harvest. Nature..

[101] Mongraw-Chaffin ML, Anderson CA, Allison MA, Ouyang P, Szklo M, Vaidya D (2015). Association between sex hormones and adiposity: qualitative differences in women and men in the multi-ethnic study of atherosclerosis. J Clin Endocrinol Metab.

[102] Mathur P, Ostadal B, Romeo F, Mehta JL (2015). Gender-related differences in atherosclerosis. Cardiovasc Drugs Ther.

[103] Liou AP, Paziuk M, Luevano JM, Machineni S, Turnbaugh PJ, Kaplan LM (2013). Conserved shifts in the gut microbiota due to gastric bypass reduce host weight and adiposity. Sci Transl Med.

